# Toddlers Viewing Fantastical Cartoons: Evidence of an Immediate Reduction in Endogenous Control Without an Increase in Stimulus‐Driven Exogenous Control

**DOI:** 10.1111/desc.70008

**Published:** 2025-03-16

**Authors:** Claire Essex, Rachael Bedford, Teodora Gliga, Tim J. Smith

**Affiliations:** ^1^ Centre for Brain and Cognitive Development, Birkbeck University of London London UK; ^2^ Department of Psychology, Queen Mary University of London London UK; ^3^ University of East Anglia Norwich UK; ^4^ Creative Computing Institute University of the Arts London London UK

**Keywords:** endogenous attention control, executive function, exogenous attention control, fantastical content, toddlers TV viewing

## Abstract

Empirical studies have shown immediate detrimental effects of TV viewing on children's executive functions (EFs). Existing theories of TV viewing have proposed that such depletion could occur due to fantastical cartoons triggering an attention bias towards salient features of the stimuli (e.g., stimulus‐driven exogenous attention). However, a co‐occurrence of salient visual features known to drive attention exogenously in fantastical cartoons means it is unclear which aspect of the content is problematic. In the present study, we matched clips on visual saliency to isolate and test the short‐term impact of fantastical content. Specifically, we tested (1) performance on an inhibitory control (IC) task (a gaze‐contingent anti‐saccade task) as a measure of EF depletion, whilst 36 toddlers (18 months) viewed cartoons with and without fantastical events (7‐min viewing duration), and (2) whether differences in IC are associated with increased stimulus‐driven exogenous attention. Results confirmed an immediate detrimental effect of fantastical cartoons on toddlers’ endogenous control (indexed by anti‐saccade behaviours), with toddlers less able to inhibit looks to a distractor to make anticipatory looks to a target. However, fixation durations (FDs) during cartoon viewing and speed of orienting to a distractor on the anti‐saccade task did not differ between the two viewing conditions, suggesting no effects on exogenously driven attention. These results point to a detrimental impact of fantastical cartoons on endogenous control mechanisms, which may have arisen from cognitive processing difficulties.

## 1 Introduction

Executive functions (EFs) are a set of cognitive control processes that allow us to deploy our attention and behaviour in a goal‐directed manner (Miyake et al. 2000). EFs have been associated with children's success at school and later in life (Blair and Razza 2007; Gathercole et al. 2004). The prominent developmental model of EFs proposed by Diamond (2013) specifies three core components: *inhibition* (or inhibitory control [IC]; behavioural and cognitive interference control [selective attention and cognitive inhibition]), *working memory (WM)* and *cognitive flexibility (CF)*. In adults, performance on executive function tasks is driven by a common EF factor (primarily associated with IC) and separable updating‐specific and shifting‐specific factors (e.g., showing a pattern of unity and diversity; Miyake and Friedman 2012). In the developmental literature, the evidence is less conclusive. Some studies have shown that performance on executive function tasks may best be explained by a single latent executive function construct (e.g., Brydges et al. 2014; McKenna et al. 2017; Wiebe et al. 2008; Wiebe et al. 2011) whilst others have found evidence of dissociable factors in children from approximately two years of age (e.g., Bernier et al. 2012; Garon et al. 2014; Mulder et al. 2014; Skogan et al. 2015).

IC is the ability to override an automatic prepotent response by controlling one's attention, behaviour, thoughts and/or emotions (Diamond 2013). In the present study, we focus on the IC of attention, defined as the ability to selectively attend based on internal goals (e.g., endogenous, top‐down control) whilst suppressing prepotent/automatic responses to a conflicting salient stimulus (e.g., stimulus‐driven exogenous attention). Although predominantly under genetic control, it has been suggested that the prolonged development of cortical regions supporting EFs and attention control more broadly makes them susceptible to environmental influences (Posner et al. 2016; Bernier et al. 2012; Zelazo and Carlson 2012). One environmental factor which is thought to influence EFs and attention control is screen media (Dye and Bavelier 2010; Nikkelen et al. 2014; Rothbart and Posner 2015; Rueda et al. 2005).

Screen media permeates children's everyday lives, from entertainment at home, educational videos online and in the classroom, to convenient app‐based presentations on mobile devices (e.g., smartphones and tablets). Interactions with screen media begin early in life (Bedford et al. 2016; Panjeti‐Madan and Ranganathan 2023; Rideout and Robb 2020), increase across childhood (Goode et al. 2020; Rideout and Robb 2020; Ofcom 2023) and have been linked with several negative developmental outcomes including emerging attention problems (e.g., inattentiveness, hyperactivity and impulsivity; Nikkelen et al. 2014). Although evidence remains equivocal, the potential negative consequences of viewing screen media have led to a broad consensus amongst leading health organisations (e.g., American Academy of Paediatrics 2016; World Health Organisation 2019) that “screen time” during early childhood should be limited and monitored by caregivers. However, a monolithic approach to screen time likely misses the nuance of children's interaction with screen media. A variety of factors may mediate any potential adverse effects, such as the age of first exposure, duration of exposure, interactivity of exposure (e.g., TV/Video Viewing, gaming, educational apps), nature of the content viewed and individual characteristics of the viewer and their environment (temperament, household SES, etc.). Understanding the mechanisms by which negative consequences of media viewing may emerge continues to be of the utmost importance for researchers, parents and practitioners.

**Summary**

- After viewing fantastical cartoons, toddlers show reduced endogenous control, indexed by fewer anticipatory looks on an anti‐saccade task.
- Matching the visual demands of cartoons with and without fantastical events revealed that the effects on endogenous control are not due to differences in visual saliency.
- Measures of stimulus‐driven exogenous attention *during* cartoon viewing did not differ between cartoons with and without fantastical events.
- We extend empirical evidence of the impact of TV viewing on toddlers under two and suggest the need to investigate mechanisms underlying cognitive fatigue.

### 1.1 Short‐Term Impact of TV Viewing—Understanding Mechanisms

Experimentally testing the direct short‐term effects of different types of content is a first step towards identifying possible mechanisms which could impact longer‐term development via prolonged, repeated exposure. Across multiple studies, EFs have been shown to be impaired immediately after viewing particular types of children's TV shows but not others (Huber et al. 2018; Kostyrka‐Allchorne et al. 2019; Li et al. 2020; Lillard, Drell et al. 2015; Lillard and Peterson 2011; Rhodes et al. 2020). Although early work examined the role of pacing (e.g., frequency of onscreen audio‐visual changes; Anderson et al. 1977; Cooper et al. 2009; Huston‐Stein et al. 1981; McCollum Jr. and Bryant 2003), a thorough investigation of TV viewing by Lillard and Peterson (2011) and Lillard and colleagues (2015) identified fantastical content, irrespective of pacing, as a key ingredient.

Lillard and colleagues (2015) defined fantastical content as containing characters or objects which undergo impossible physical or identity transformations or exhibit impossible attributes such as violations of continuity (e.g., Road Runner running through a picture of a tunnel as if it was real). Fully crossing *Pace* (fast/slow) × *Content* (fantastical/non‐fantastical) between‐clip conditions, they found that EF performance was poorer only after viewing fantastical TV content, with no effect of *Pace* and no interaction between *Pace* × *Content*. Although a lack of pre‐viewing measures of EFs limited the interpretation of the initial findings, multiple studies with pre‐viewing measures of EFs have since replicated the findings (Rhodes et al. 2020; Huber et al. 2018; Li et al. 2020). These studies have shown a detrimental impact of viewing fantastical content on dimensions of inhibition (e.g., cognitive interference control, motor inhibition, behavioural inhibition), WM and CF in children between 2 and 6 years.

Lillard, Li et al. (2015) suggested the impact on EF performance may have been due to increased use of exogenous attention driven by stimulus factors (e.g., when attentional deployment is driven purely by external factors reflecting sensory stimulation; Corbetta and Shulman 2002), with frequent re‐orienting during viewing thought to quickly exhaust the attentional resources needed for performance on the EF tasks after viewing. A study by Li et al. (2020) appears to confirm this increase in exogenous attention whilst children aged 4–6 years viewed fantastical content. Measuring gaze behaviour during viewing, they found shorter mean fixation durations (FDs) and a greater number of fixations for children who viewed highly fantastical content than children who viewed content low on fantastical events. A pattern, they argued, which was consistent with the frequent re‐orienting proposed by Lillard, Li et al. (2015). The same children subsequently performed less well on behavioural measures of EFs than children who viewed content rated as low on fantastical events.

However, it is important to note that most prior studies have compared different clips across conditions, conflating the inherent difference in audio‐visual features between clips with the conditions (e.g., pacing or fantasy differences). Essex et al. (2022) tracked key featural/semantic properties in the clips used by Lillard and Peterson (2011) and Lillard, Drell et al. (2015) and found a higher prevalence of visual features which contribute to overall saliency (e.g., flicker and edge density) in the TV shows that had detrimental impacts on children's EFs. These features drive attention in a bottom‐up fashion, amplified by structural properties such as cuts (e.g., transitions between shots), to guide viewers towards areas of high salience in any given shot (Carmi and Itti 2006). Thus, the results of Essex et al. (2022) raise the possibility that saliency differences rather than fantastical events per se may have negatively impacted EFs. However, to date, there has been a lack of research seeking to measure this potential (saliency or fantasy‐driven) exogenous bias during viewing. With the present study, we therefore sought to test the exogenous bias hypothesis put forward by Lillard, Li et al. (2015) and to confirm this is triggered by the surprising nature of the fantastical events by ensuring saliency differences which impact exogenous attention (Carmi and Itti 2006) are controlled in the chosen video clips.

In addition, we further build on existing evidence by extending the investigation of short‐term effects of TV viewing to children under 2 years of age. Evidence suggests that from approximately four months of age, children can anticipate trajectories of object paths on a computer screen (Johnson et al. 2003), display knowledge of object properties such as object permanence (Baillargeon 2008; Bremner et al. 2015) and spatiotemporal continuity (Spelke et al. 1992; Wynn 1995). From 6 months of age, infants can also distinguish between predictable and unpredictable action sequences and use this information to anticipate the goal of an action, as reflected by differences in anticipatory gaze behaviours (Geangu et al. 2015; Kochukhova and Gredebäck 2010; Hunnuis and Bekkering 2014; Baldwin et al. 2001). However, their ability to interpret and integrate such information as part of a screen‐based narrative appears to develop slowly over the preschool years (Ildirar Kirbas and Smith 2018). For example, whilst older children will rationalise about narrative moments which deviate from reality through the maintenance of schema specific to the narrative context (e.g., SpongeBob's sponge body facilitates his impossible body transformations), younger children are not yet able to do so (Ildirar Kirbas and Smith 2018). That said, by approximately 18 months of age, children are beginning to display sufficient perceptual skills to detect violations of basic situational continuity factors in TV content (e.g., toddlers can distinguish between shuffled and unshuffled content; Pempek et al. 2010), but cannot yet resolve these violations based on the background narrative, making this a key age for examining the influence of fantastical events.

### 1.2 Current Study

In the current study, two sets of cartoon stimuli were created with and without fantastical events, matched on visual demands at the level of visual salience. A detailed description of the cartoon stimuli with saliency analysis is provided in Section 2.3. We first sought to replicate existing EF depletion findings with a toddler‐friendly IC task (the Anti‐Saccade task). The anti‐saccade task measures the suppression of automatic saccades to a salient distractor with concomitant execution of anticipatory saccades towards a contralateral target location. It is, therefore, possible to index the competing interplay between exogenous (e.g., automatic saccades to a distractor; *pro‐saccades*) and endogenous control (e.g., inhibition of distractor with anticipatory saccades to target; *anti‐saccades*). In the adult anti‐saccade task, attempts to resolve conflict between competing stimuli typically lead to slower response times and increased error rates (Diamond 2013). In early childhood, modified versions of the anti‐saccade task serve as a key measure of IC (Johnson 1995; Scerif et al. 2005; Portugal, Bedford, Cheung, Mason et al. 2021). Unlike older children and adults, young children cannot be instructed to ignore the presence of a peripheral distractor and to look towards a target location. As such, in the adapted anti‐saccade task, children are encouraged to suppress their looks to the distractor by issuing a motivating reward at the target location. Evidence suggests the ability to suppress automatic saccades to a peripheral distractor is present from 4‐months‐of‐age (Johnson 1995), whilst the production of anticipatory saccades to the target emerges during the second year of life and improves across the toddler years (Scerif et al. 2005). Extending prior findings, the anti‐saccade task was inserted between blocks of cartoon viewing, allowing us to assess the impact over time. In this toddler‐friendly version of the anti‐saccade task (Johnson 1995; Scerif et al. 2005; Portugal, Bedford, Cheung, Mason et al. 2021), the suppression of automatic responding with concomitant anticipation of the target is learnt over the course of the trials, leading to an increase in anti‐saccade behaviour and corresponding decreases in pro‐saccades (to the distractor) and corrective‐saccades (fast pro‐saccades followed by anticipatory saccades to the target; Portugal, Bedford, Cheung, Mason et al. 2021). However, if fantasy viewing has a detrimental impact on IC, we would expect this learning effect to be reduced when children view cartoons **with fantastical events** compared to when cartoons **without fantastical events** are viewed.

In line with the EF depletion literature, we hypothesised that viewing cartoons **with fantastical events** would lead to poorer performance on the IC task, as indexed by reduced anti‐saccade behaviour. To test whether exogenous attention increased *during* viewing, we assessed FDs during cartoon viewing. In line with the findings by Li et al. (2020), we expected increased exogenous attention during viewing to lead to shorter FDs.

## 2 Methods

### 2.1 Participants

Thirty‐six toddlers (18 girls) aged 18 months (Mean: 564 days, SD: 12) were recruited between April 2021 and April 2022 through the Birkbeck Babylab database and social media channels. Children were from predominantly highly educated families (University degree or above, *N* = 35), living in Central London. Families visited the Babylab twice, 1 week apart (mean: 9 days, SD: 5). Before the first lab visit, a time‐use diary was administered to capture children's media habits over a 24‐h period. Parents were asked to provide retrospective reports of their child's daily activities for each hour of their chosen day (e.g., sleeping/resting, eating/drinking, nursery/daycare, bathroom/grooming, indoor play/recreation, outdoor play/recreation, media use and travelling). If parents indicated media use, they were then asked a series of follow‐up questions to capture the nature and context of the media use. The average total media use (e.g., all media use [video content, music, games, creative apps, internet‐based searching] across all devices [TV, mobile devices, computers, games consoles]) over the 24‐h period, was 55 min (SD: 53 min). The full sample details are reported in Supporting Material S1. The study was approved by the Birkbeck Psychological Sciences ethics board and conducted according to the British Psychological Society Code of Ethics and Conduct. Parents provided written informed consent for their child at the first visit.

### 2.2 Study Design

**FIGURE 1:**
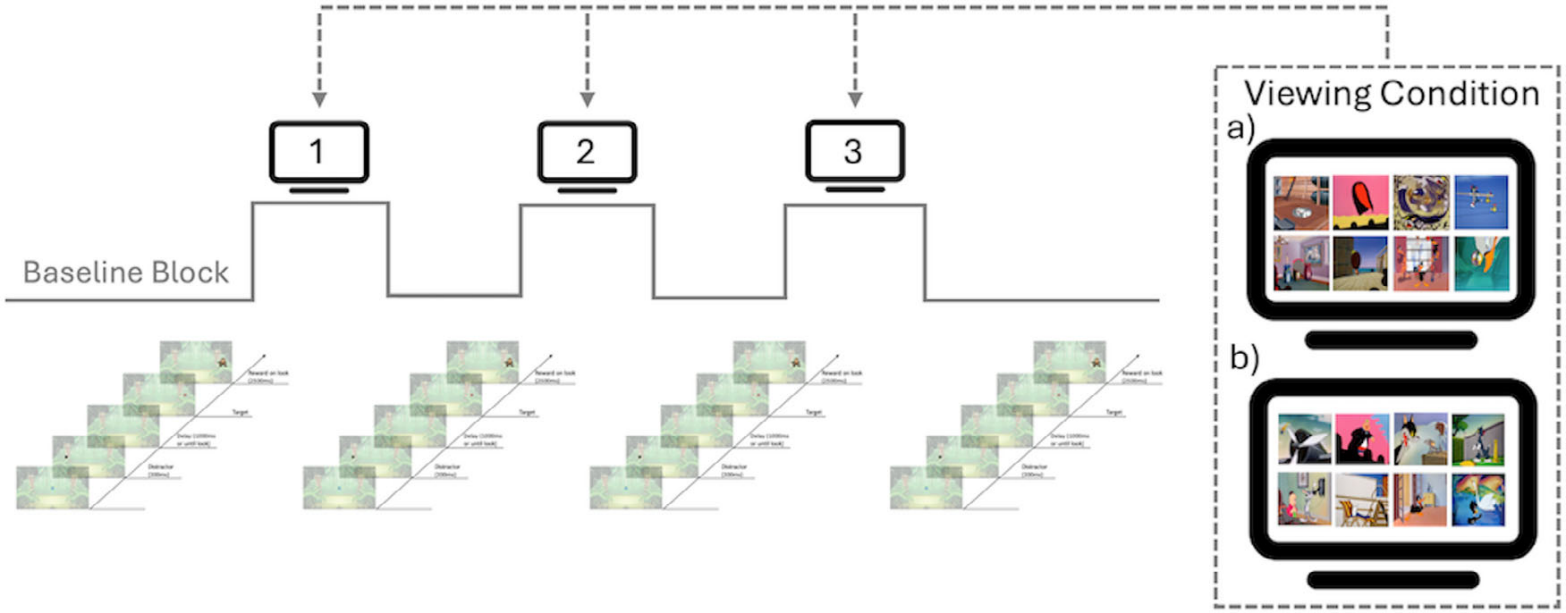
Schematic of the experimental procedure. Cartoon stimuli with (a) or without fantastical (b) events were presented three times between blocks of the anti‐saccade task. The first block of the anti‐saccade task served as a baseline block.

The study utilised a within‐subjects block design with children completing two experimental viewing conditions (with/without fantastical events) across the two visits. The eye‐tracking‐based IC task was administered in a block design between blocks of cartoon viewing to capture ongoing measures of saccade behaviour throughout the cartoon viewing. For the IC task, there were two within‐subjects independent variables: (1) viewing condition (with/without fantastical events) and (2) block (2–4). For the cartoon viewing, there were also two within‐subjects independent variables: (1) viewing condition (with/without fantastical events) and (2) block (1–3). A schematic of the block design is provided in Figure 1. These tasks were administered as part of a larger battery of tasks, including additional eye‐tracking paradigms and a behavioural play task that the present study did not consider. The entire task battery can be found in Supporting Material S2. The total session duration was ∼25 min.

### 2.3 Cartoon Stimuli

**FIGURE 2:**
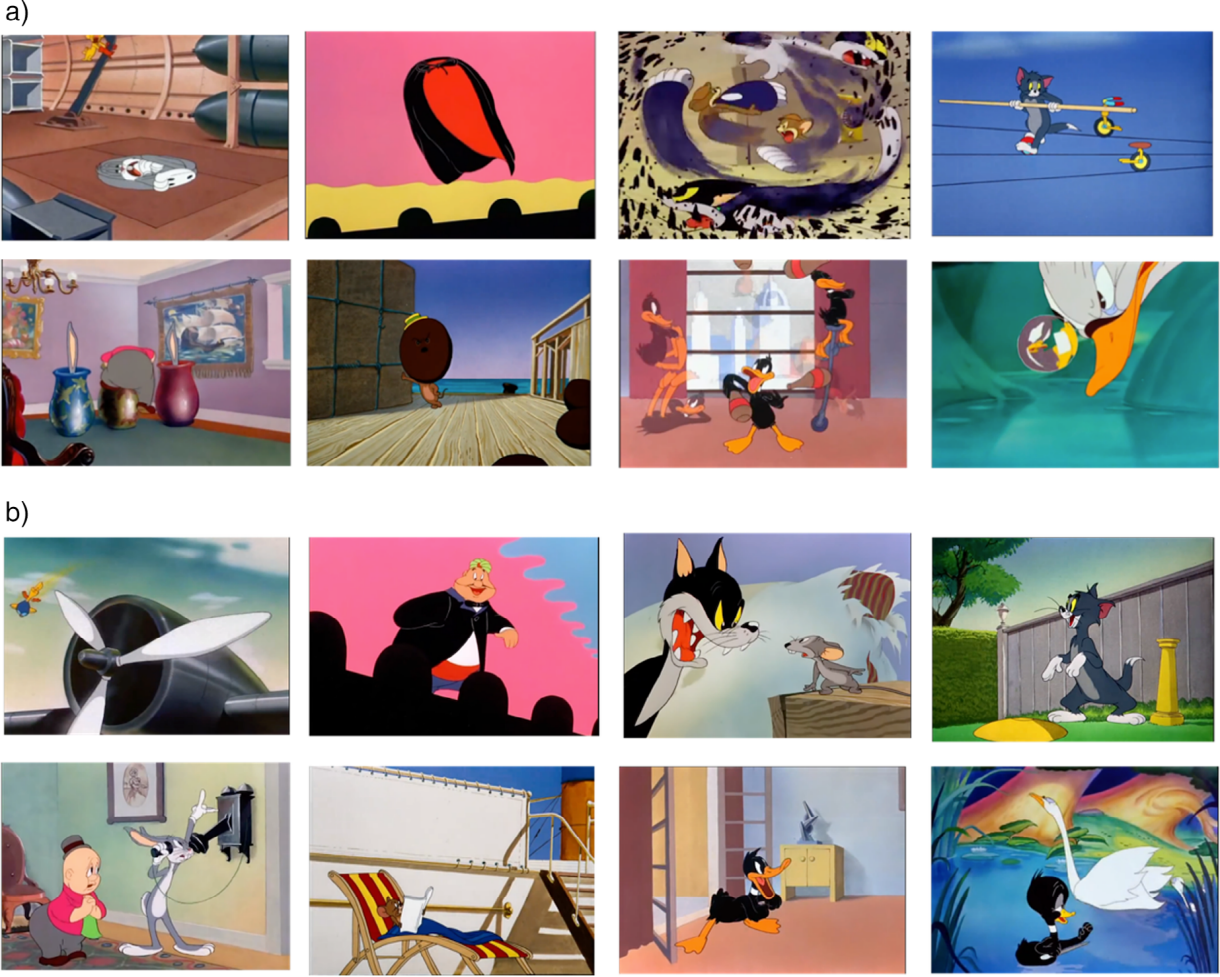
Stills from the (a) with fantastical events and (b) without fantastical events matched cartoon content.

To test the short‐term impact of fantastical cartoon content, we created two sets of experimental videos (with/without fantastical events) shown in Figure 2a,b. The videos contained eight clips from Looney Tunes cartoons (1930–1969), with a fantastical clip matched to a clip without a fantastical event from the same episode. Each fantastical clip contained one fantastical event (i.e., events depicting violations of continuity, cohesion, solidity, etc.). For example, Daffy Duck violates the law of cohesion by appearing to multiply to perform a dance routine. Looney Tunes episodes received a U‐rating from the British Board of Film Classification (BBFC), indicating they are universally suitable for all ages. Clips were matched on duration, characters and cut frequency (see Table 1). Cross‐dissolves and matched‐action cuts were used to minimise disruption from transitions between clips. Thus, clips followed editing techniques commonly used in TV/Film to hide cuts (Smith 2012). To avoid conflating the challenge of processing fantastical events with general narrative complexity, all clips were taken from semantically coherent sequences and contained minimal dialogue. Video stimuli are accessible via the Open Science Framework (OSF; https://osf.io/vadqx/).

**TABLE 1:** Summary of features present in the two sets of cartoon stimuli.

Feature	With fantastical events	Without fantastical events	Wilcoxon signed‐rank test
Total video duration	2 min 18 s	2 min 18 s	
Characters (*N*)	16	16	
Locations (*N*)	8	10	
Situational change	88%	84%	
Total number of cuts (as cuts *p*/min)	19 (8.26)	14 (6.09)	n.s. (0.24)
Low‐level saliency (flicker)	2,118,883.95 (1,062,221.17)	1,715,816.98 (1,059,173.78)	n.s. (*p* = 0.48)
Event‐based low‐level saliency (flicker)	2,456,804.86 (1,594,759.19)	1,826,212.00 (1,217,966.99)	n.s. (*p* = 0.33)

*Note*: Continuous measures (e.g., durations, Total number of cuts, low‐level saliency [flicker], event‐based low‐level saliency [flicker]) are presented as mean (standard deviation). Flicker is measured in arbitrary units. Categorical measures (e.g., characters and locations) are presented as frequency counts. Situational change (e.g., discontinuity in time/space/action) is presented as a proportion of all edits. Paired sample contrasts were tested with the non‐parametric Wilcoxon Signed Rank Test. Other descriptive values have not been tested statistically due to contributing an insufficient number of data points.

Edited video stimuli were exported at a 1280 × 720 resolution and presented in a 16:9 aspect ratio on a 23'' widescreen monitor with stereo speakers. Due to the original 4:3 aspect ratio of the cartoons, the clips had a black border. The eight clips were presented three times in three blocks. Repetition of the clips across the blocks occurred in a pseudo‐randomised order to prevent children from anticipating the events. Children saw the cartoons **with or without fantastical events** at each visit. Viewing condition was counterbalanced across visits such that half the sample (*N* = 18) saw the cartoons **with fantastical events** at visit 1 and **without fantastical events** at visit 2, whilst the other half of the sample saw cartoons **without fantastical events** at visit 1 and **with fantastical events** at visit 2.

#### 2.3.1 Cartoon Stimuli—Saliency Analysis

A computational analysis of the stimuli was performed to confirm that we had successfully matched the conditions at the saliency level. Full details of the computational methods used have been reported previously (Essex et al. 2022). The Matlab computer vision toolbox (v 9.5) was used to compute our chosen measure of saliency, *flicker* (e.g., change in pixel luminance between frames). Videos were down‐sampled to 256 × 256 pixels to ensure videos were compared on a standard resolution. Videos were first converted into CIELab colour space, separating luminance from colour before calculating flicker frame by frame. Flicker, representing the change in luminance between corresponding pixels of adjacent frames, was computed from the normalised resolution and the full range of luminance change at each pixel. For the present analysis, mean luminance difference values were calculated for each clip and then averaged across all clips to give an average flicker for each viewing condition. A non‐parametric Wilcoxon Signed‐Rank Test showed that conditions did not differ on average flicker (*Z* = −0.700, *p* = 0.48). As fantastical events may be associated with more low‐level feature change, we also tested the saliency difference between conditions during the fantastical events. We did so by time‐matching fantastical sequences with the corresponding time frame in the matched **without fantastical events** clip. This also showed no statistical difference between the two conditions (*Z* = −0.98, *p* = 0.33). Bayesian *t*‐tests showed moderate evidence for the null hypothesis between the two sets of cartoons on both saliency measures (Supporting Material S3). Thus, the two conditions did not differ at the saliency level (See Table 1).

### 2.4 Lab Measures of Gaze Behaviours

Gaze behaviours were measured during free‐viewing of the cartoons and with a gaze‐contingent IC paradigm. Participant's eye movements were recorded at 120 HZ using a Tobii TX300 eye‐tracker, MATLAB and the Tobii Analytics SDK on a MacBook Pro. Stimuli were presented on a 23'' widescreen monitor at an aspect ratio of 16:9 (1920 × 1080 pixels) with stereo speakers via custom scripts using PsychToolbox (version 3.0.12). Children were seated on their caregiver's lap 60 cm from the screen. Participants’ gaze was calibrated using a child‐appropriate 5‐point procedure (Senju and Csibra 2008) at the start of the session. After calibration, the stimulus presentation ran automatically (the pacing of trials and timing of stimulus presentation were contingent on the child's gaze). During the free‐viewing of the cartoons, the stimulus presentation continued until the end of the video. On the IC task, the presentation of the stimulus was contingent on the child's gaze at the start of each trial. If the child's gaze did not trigger the gaze‐contingent stimulus (due to fussiness or loss of gaze), trials were triggered manually by the experimenter and automatically flagged as invalid. The session was monitored and recorded with a participant‐facing web camera located above the display screen and ScreenFlow (Telestream Inc., version 9.0) screen‐casting software.

#### 2.4.1 Cartoon Viewing

Spontaneous eye movements were captured whilst children viewed the cartoon clips. Each cartoon block was triggered automatically and was presented continuously for a total duration of 2 min and 18 s. Fixation parsing was performed using Gazepath (v.1.21; van Renswoude et al. 2018) in RStudio (v 4.2.2; RStudio Team 2022). Gazepath parses the raw eye‐tracking data into fixations (e.g., the period between two saccadic eye movements) and saccades (e.g., the period in which eyes are in flight). It was designed to identify fixations in infant data, which is notoriously noisy (Wass et al. 2013), whilst accounting for individual differences in data quality. To hold parameters constant within participants, the session data (e.g., a maximum of two session data) was parsed participant by participant. Gazepath automatically dropped FDs below 100 ms. Further cleaning of individual fixations was performed after parsing to identify improbably long fixations (>3000 ms), which were cleaned out of the data prior to analysis. The outcome measure during video viewing was calculated as **mean FDs** averaged across all fixations (e.g., sum individual FDs / total number of fixations) for each block in each viewing condition (with/without fantastical events). To account for differences in data quality, the proportion of parsed samples with gaps arising from lost gaze was calculated (e.g., total number of parsed samples with gaps/total number of valid parsed samples). No further inclusion/exclusion criteria were applied.

#### 2.4.2 Anti‐Saccade IC Task

**FIGURE 3:**
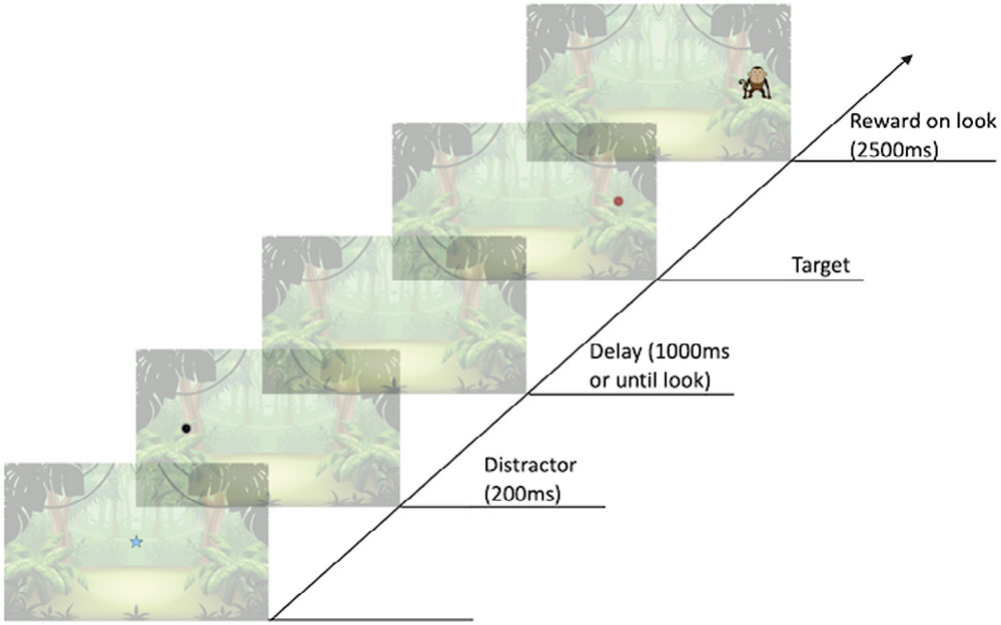
Trial schematic for the anti‐saccade task. Every trial started with a gaze‐contingent central fixation stimulus. Upon fixation of the central stimulus, a distractor stimulus (black circle) appeared for 200 ms on one side of the screen. After 1000 ms (or until look), a target stimulus (red circle) was presented on the opposite side of the screen. When the child looked at the target location, a reward stimulus (an animated animal with accompanying sound effects) was presented for 2500 ms. The delay between distractor offset and target onset provided the time window for successful anticipation of the target. Stimuli are drawn to scale.

The Anti‐Saccade IC task was presented in four blocks. The first block served as a baseline block and was presented immediately before the first video block. Each subsequent block was presented immediately after a video block. Figure 3 shows the stimulus sequence for the task. Each block contained 15 trials, beginning with a gaze‐contingent central fixation star (subtending 3° × 3°). Immediately after the fixation of the central stimulus (CS) area of interest (AOI), a distractor stimulus (DS; black circle, subtending 3° × 3°) was presented for 200 ms to the left or right of the screen (17° eccentricity). 1000 ms after the distractor offset, a target stimulus (TS; red circle, subtending 4° × 4°) was presented on the opposite side of the screen (17° eccentricity). The delay between distractor offset and target onset (as indicated in Figure 3) provided the time window for successful anticipation of the target (e.g., anti‐saccade). Upon target fixation, a reward stimulus (cartoon animals, subtending 8° × 8°) with audio replaced the red TS for 2500 ms, and the trial ended. The reward animation was issued immediately if the child looked at the target before the target onset. The side of the target and distractor did not switch within the visit, but the side was counterbalanced across visits.

If the child did not look at the screen, the experimenter manually presented an audio attention‐getter (jingle sound) to draw the gaze back to the screen. If the child's gaze did not land on the CS and automatically trigger the trial (validity criteria 1), the researcher could manually start a trial and mark it as invalid. Such manual trial rejections occurred in 4.17% of total trials and were generally due to poor tracking, child fussiness or non‐compliance with the task, for example, failing to look back to the centre between trials. Other offline validity criteria were applied to ensure consistency in data quality and the starting point of saccades contributing to the analysis of looks and reaction times. Trials were considered invalid if: (validity criteria 2) the child did not fixate on the target location at any point in the trial (1.86% of total trials), (3) there was too much missing data between distractor onset and gaze in the target AOI (≥100 ms; occurring on 7.77% of total trials), (4) gaze was not in the CS AOI at distractor onset (0.39% of total trials), (5) gaze was found in the distractor AOI prior to DS onset (0.44% of total trials), or (6) gaze was found in the target AOI prior to DS onset (0.47% of total trials). Only valid trials were considered for further computation of the outcome measures. Supporting Material S7 presents the mean number of valid trials obtained for each viewing condition as a function of block. Paired‐comparison *t‐*tests for each block confirmed that validity did not differ between the two viewing conditions (all *p* > 0.05).

The location of looks and reaction times in each trial were measured offline according to the following look classifications: (1) a look to the distractor without looking at the target was classified as a ***pro‐saccade***; (2) an anticipatory look to the target (e.g., before, or within 100 ms of target onset) without a saccade to the distractor was classified as an ***anti‐saccade***; (3) a saccade to the distractor followed by an anticipatory saccade to the target was classified as a ***corrective saccade***.

For each participant, the proportion of each saccade behaviour (pro‐saccade, anti‐saccade and corrective saccade) was calculated for each block (1–4) in each viewing condition (with/without fantastical events). This was calculated as the number of pro, anti and corrective saccades (saccade behaviours were mutually exclusive in a trial) divided by the total number of valid trials. Only participants with at least five valid trials were included in the analysis (this was based on the distribution of valid trials; see Supporting Materials S4 and S5).

### 2.5 Analytic Approach

All data preprocessing and analyses were performed in Matlab, R and SPSS. Generalised estimating equations (GEE; Liang and Zeger 1986) were used to test condition differences during cartoon viewing and the IC task. This approach is increasingly being used in the developmental literature for studies employing a repeated measures design, to avoid reducing the sample size due to data loss at individual data points (Muth et al. 2016; Portugal, Bedford, Cheung, Gliga et al. 2021; Portugal, Bedford, Cheung, Mason et al. 2021; Woythaler et al. 2011; Vernetti et al. 2018; Lockwood Estrin et al. 2024). A linear model with an identity link and unstructured correlation matrix was used for all analyses to predict outcome measures.

For the anti‐saccade task, separate GEE models were performed for the three mutually exclusive saccade behaviours (e.g., the proportion of anti‐, pro‐ and corrective saccades). Two within‐subjects variables were entered as predictors of the outcome variable: *viewing condition* (with/without fantastical events) and *block* (2–4). An exploratory analysis of the latency to look to the distractor was performed to check whether differences in saccade behaviours were explained by differences in the speed of responding to the distractor. *Viewing condition* (with/without fantastical events) and *block* (2–4) were entered as within‐subjects predictors of the outcome variable (latency to look to the distractor). The dependent variables for the anti‐saccade task were proportion of anti‐saccades, pro‐saccades, corrective saccades and distractor latency (e.g., saccadic reaction time). All measures were baselined to control for pre‐viewing differences by subtracting the value in block 1 (pre‐viewing) from the value in each subsequent block.

To measure exogenous attention during cartoon viewing, the outcome variable was mean FD (ms). *Viewing condition* (with/without fantastical events) and *block* (1–3) were entered as the within‐subjects predictors of the outcome variable.

## 3 Results

### 3.1 Performance in the Anti‐saccade Task Is Influenced by the Presence of Fantastic Events

**FIGURE 4:**
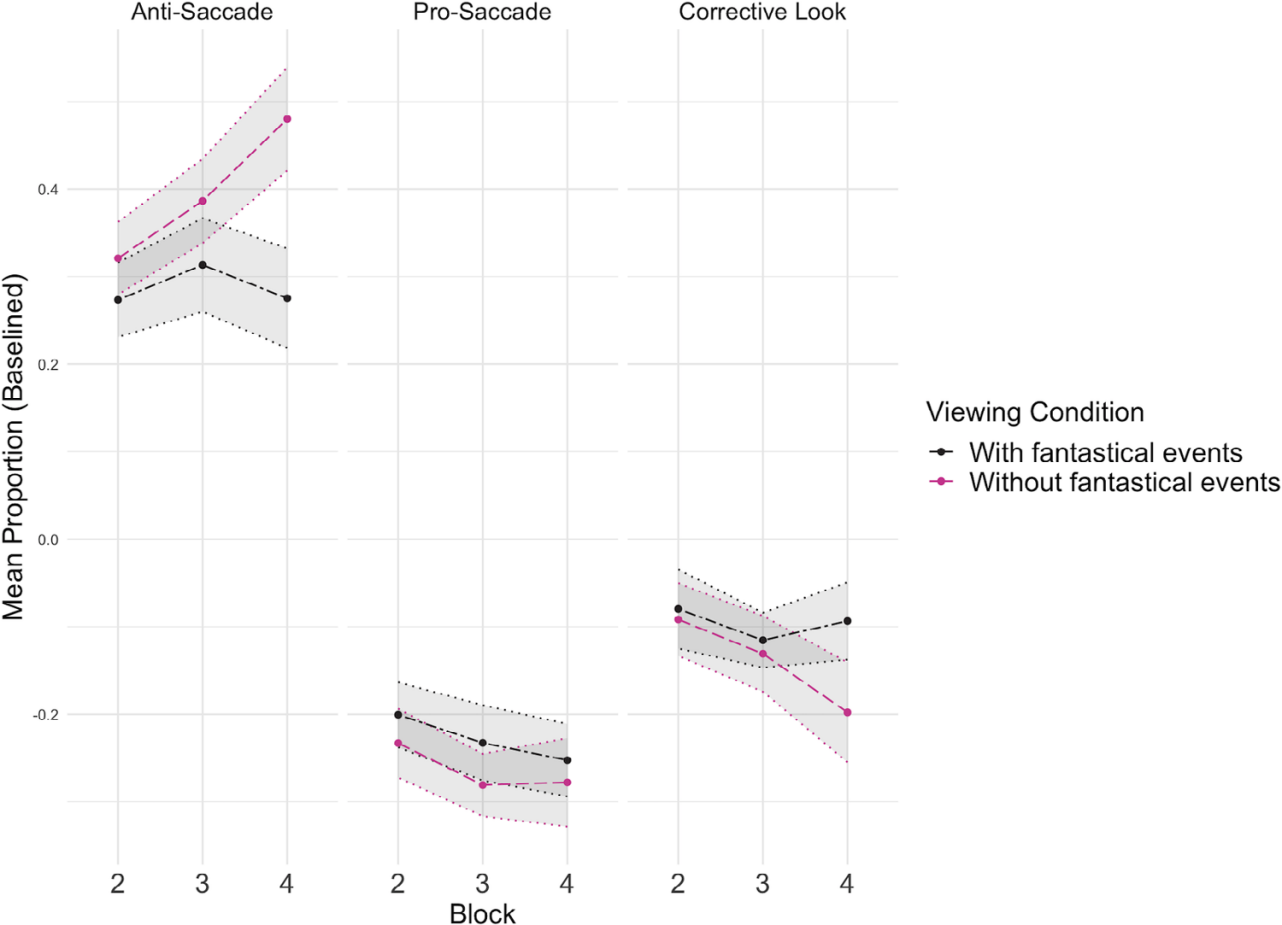
Mean difference in the proportion of saccade behaviour relative to block 1 for each viewing condition as a function of block and saccade behaviour in the Anti‐Saccade Task. Shaded areas represent the standard error of the mean.

Initial GEE models with *viewing conditions* (with/without fantastical events) entered as predictors of saccade behaviour (anti‐saccade, pro‐saccade, corrective saccade) in the baseline block confirmed conditions were matched at baseline (Supporting Material S6). For data quality purposes, a GEE model with *block* (2–4; controlling for baseline‐Block 1) and *viewing condition* (with/without fantastical events) entered as predictors of the number of valid trials was conducted. This showed a significant effect of block (Wald *x* ^2^ = 7.15 (1), *p* = 0.03). The number of valid trials decreased with block and was, therefore, included as a covariate for all subsequent analyses. This did not differ between viewing conditions (Supporting Material S8). The results for saccade behaviours are shown in Figure 4. The full GEE model results for saccade behaviours are shown in Table 2. See Supporting Material S9 for saccade behaviours included in the analysis without baseline correction. Descriptive statistics for all outcome measures are shown in Table 3.

**TABLE 2:** Summary of generalised estimating equations model effects with block and viewing condition as predictors of outcome measures.

Variables	Wald ×2 (df), *p* value
Main model with block and viewing condition as predictors of saccade behaviour (controlling for number valid trials)	
***Anti‐saccades (as proportion of total valid trials)***	
**Block**	**7.266 (2), *p* = 0.03**
Viewing condition	2.968 (1), *p* = 0.09
*Valid trials*	*1.56 (1), p = 2.11*
Block* viewing condition	**6.281 (2), *p* = 0.04**

Follow‐up model: with fantastical events viewing condition	
Block	2.189 (2), *p* = 0.34

Follow‐up model: without fantastical events viewing condition	
**Block**	**9.787 (2), *p* = 0.007**

Follow‐up model: Block 2	
Viewing condition	0.112 (1), *p* = 0.74

Follow‐up model: Block 3	
Viewing condition	1.122 (1), *p* = 0.29

Follow‐up model: Block 4	
**Viewing condition**	**4.103 (1), *p* = 0.04**
***Pro‐saccades (as proportion of total valid trials)***	
**Block**	**7.236 (2), *p* = 0.03**
Viewing condition	0.804 (1), *p* = 0.37
*Valid trials*	*0.609 (1), p = 0.44*
Block* viewing condition	0.366 (2), *p* = 0.83
***Corrective looks (as proportion of total valid trials)***	
**Block**	**5.344 (2), *p* = 0.07**
Viewing condition	0.225 (1), *p* = 0.64
*Valid trials*	*3.561 (1), p = 0.06*
Block* viewing condition	1.416 (2), *p* = 0.49

Follow‐up model: with fantastical events viewing condition	
Block	1.288 (2), *p* = 0.53

Follow‐up model: without fantastical viewing condition	
**Block**	**6.611 (2), *p* = 0.04**

Follow‐up model: Block 2	
Viewing condition	0.179, (1), *p* = 0.67

Follow‐up model: Block 3	
Viewing condition	0.024 (1), *p* = 0.88

Follow‐up model: Block 4	
Viewing condition	0.49, (1), *p* = 0.49
***Latency to distractor (saccadic reaction time)***	
Block	3.373 (2), *p* = 0.19
Viewing condition	1.370 (1), *p* = 0.24
***Valid Trials***	***7.329 (1), p = 0.007*** ^a^
Block* viewing condition	4.012 (2), *p* = 0.13

*Note*: Significant results are shown in bold. Covariates are indicated with italicised text.

^a^ The average change in the outcome variable is associated with a negative change in the co‐variate e.g., longer distractor latencies are associated with fewer valid trials.

**TABLE 3:** Descriptive statistics of the outcome measures for the anti‐saccade tasks and cartoon viewing.

			With fantastical events		Without fantastical events	
**Task**	**Measure**	**Block**	***N***	**Mean (SD)**	***N***	**Mean (SD)**
Anti‐saccade	Anti‐saccade (proportion)	1	34	0.31 (0.23)	31	0.26 (0.27)
		2 (baselined)	32	0.28 (0.24)	31	0.31(0.24)
		3 (baselined)	31	0.31 (24)	30	0.31 (0.28)
		4 (baselined)	26	0.28 (0.29)	25	0.45 (0.31)
	Pro‐saccade (proportion)	1	34	0.36 (0.24)	31	0.39 (0.27)
		2 (baselined)	32	−0.19 (0.21)	31	−0.21 (0.22)
		3 (baselined)	31	−0.21 (0.21)	30	−0.22 (0.21)
		4 (baselined)	26	−0.22 (0.21)	25	−0.28 (0.23)
	Corrective look (proportion)	1	34	0.27 (0.19)	31	0.29 (0.25)
		2 (baselined)	32	−0.10 (0.23)	31	−0.09 (0.24)
		3 (baselined)	31	−0.09 (0.24)	30	−0.12 (0.19)
		4 (baselined)	26	−0.12 (0.19)	25	−0.16 (0.30)
	Distractor latency (ms)	1	33	422.76 (62.50)	31	459.26 (77.73)
		2 (baselined)	30	−5.29 (74.77)	29	17.34 (94.52)
		3 (baselined)	30	5.37 (75.93)	24	−12.69 (109.40)
		4 (baselined)	24	6.76 (80.34)	19	−12.33 (80.43)
Cartoon viewing	Fixation durations (ms)	1	36	491.24 (78.25)	33	486.36 (85.47)
		2	35	474.49 (98.07)	33	478.51 (71.64)
		3	34	479.03 (69.05)	29	460.21 (74.67)

*Note*: Saccade behaviours on the anti‐saccade task are presented as the mean proportion (standard deviation) as a function of block and viewing conditions. Fixation Durations during cartoon viewing are presented as the mean fixation duration (standard deviation) in milliseconds as a function of block and viewing conditions.

A GEE model with *viewing condition* and *block* entered as predictors of the *proportion of anti‐saccades* showed a significant effect of block (*p* = 0.03, see Table 2), with a higher proportion of anti‐saccades made in block 4 (estimated marginal mean 0.39, SE 0.04) than in block 2 (estimated marginal mean 0.30, SE 0.03), confirming that learning occurred during the task. The difference between block 2 and block 3 (estimated marginal mean 0.36, SE 0.04) and between block 3 and block 4 was non‐significant (*p* = 0.14 and *p* > 0.99, respectively). A paired *t*‐test on the proportion of anti‐saccades in block 2 and block 4, collapsed across condition, was performed to obtain an indication of the magnitude of the effect size for the main effect of block. The Cohen's d point estimate was −0.41. The proportion of anti‐saccades was higher overall in the **without fantastical events** condition (estimated marginal mean 0.40, SE 0.04) than in the **with fantastical events** condition (estimated marginal mean 0.30, SE 0.04), but this did not reach significance (*p* = 0.09). Critically, a significant block*viewing condition interaction (*p* = 0.04) showed the effect of block was driven by an increase in the proportion of anti‐saccades made in the **without fantastical events** viewing condition only (Wald *x* ^2^ = 9.79, (2), *p* = 0.007), where the proportion of anti‐saccades increased significantly from Block Two (estimated marginal mean 0.31, SE 0.04) to Block Four (estimated marginal mean 0.47, SE 0.06). The differences between block 2 and block 3 (estimated marginal mean 0.37, SE 0.04) and between block 3 and block 4 were non‐significant. Post‐hoc analysis yielded a significant difference between the conditions in Block 4 (Wald *x* ^2^ = 4.10 (1), *p* = 0.04) with a higher proportion of anti‐saccades made in the **without fantastical events** viewing condition (estimated marginal mean 0.45, SE 0.06) than in the **with fantastical events** viewing condition (estimated marginal mean 0.28, SE 0.06). A separate paired *t*‐test on the difference score between block 4 and block 2 (after controlling for baseline performance) was conducted to obtain an indication of the magnitude of the effect size. The Cohen's d point estimate was −0.20.

The alternate saccade behaviours will now be analysed to better understand the pattern of anti‐saccade proportions. For the *proportion of pro‐saccades* (e.g., automatic looks to the distractor without an anticipatory look to the target) a GEE model with *viewing condition* and *block* entered as predictors of *proportion of pro‐saccades* found no effect of viewing condition (*p* = 0.37) and no viewing condition*block interaction effect (*p* = 0.83). Block was a significant predictor of the proportion of pro‐saccades made (*p* = 0.03), with pro‐saccades decreasing across the blocks irrespective of condition. The difference between block 2 (estimated marginal mean −0.21, SE 0.03) and block 4 (estimated marginal mean −0.28, SE 0.03) was significant (*p* = 0.05). The differences between block 2 and block 3 (estimated marginal mean −0.26, SE 0.03) and between block 3 and block 4 were not significant (*p* = 0.11 and *p* = 1.00, respectively). A paired *t*‐test on the proportion of pro‐saccades in block 2 and block 4, collapsed across condition, was performed to obtain an indication of the magnitude of the effect size for the main effect of block. The Cohen's d point estimate was 0.30.

For the *proportion of corrective saccades* (e.g., saccades to the distractor followed by quick anticipatory saccades to the target), a GEE model with *viewing condition* and *block* entered as *predictors of proportion of corrective saccades* showed no effect of viewing condition (*p* = 0.64) and no viewing condition*block effect (*p* = 0.49). A marginal effect of block (*p* = 0.07) showed the proportion of corrective looks decreased from block 2 (estimated marginal mean −0.07, SE 0.03) to block 3 (estimated marginal mean −0.12, SE 0.03) only. A paired *t*‐test on the proportion of corrective‐saccades in block 2 and block 3, collapsed across condition, was performed to obtain an indication of the magnitude of the effect size for the main effect of block. The Cohen's d point estimate was 0.39. The corrective behaviours shown in Figure 4 suggest a divergence between viewing conditions at block 4. As an exploratory follow‐up, the proportion of corrective looks was tested in each viewing condition separately, with block entered as the predictor of the *proportion of corrective looks*. This showed the marginal effect of block was driven by saccade behaviour in the **without fantastical events** viewing condition only (Wald *x* ^2^ = 6.61 (2), *p* = 0.04). Where there was a marginally significant (*p* = 0.08) decrease in the proportion of corrective‐saccades from block 2 (estimated marginal mean −0.07, SE 0.04) to block 3 (estimated marginal mean −0.13, SE 0.04) and between block 2 and block 4 (estimated marginal mean −0.20, SE 0.06; *p* = 0.06). The difference between block 3 and block 4 was non‐significant (*p* = 0.37). As seen with anti‐saccade behaviour, there was no significant effect of block in the **with fantastical events** viewing condition (Wald *x* ^2^ = 1.29 (2), *p* = 0.53). The divergence in block 4 was not supported by a significant viewing condition difference (Wald *x* ^2^ = 0.49 (1), *p* = 0.49).

**FIGURE 5:**
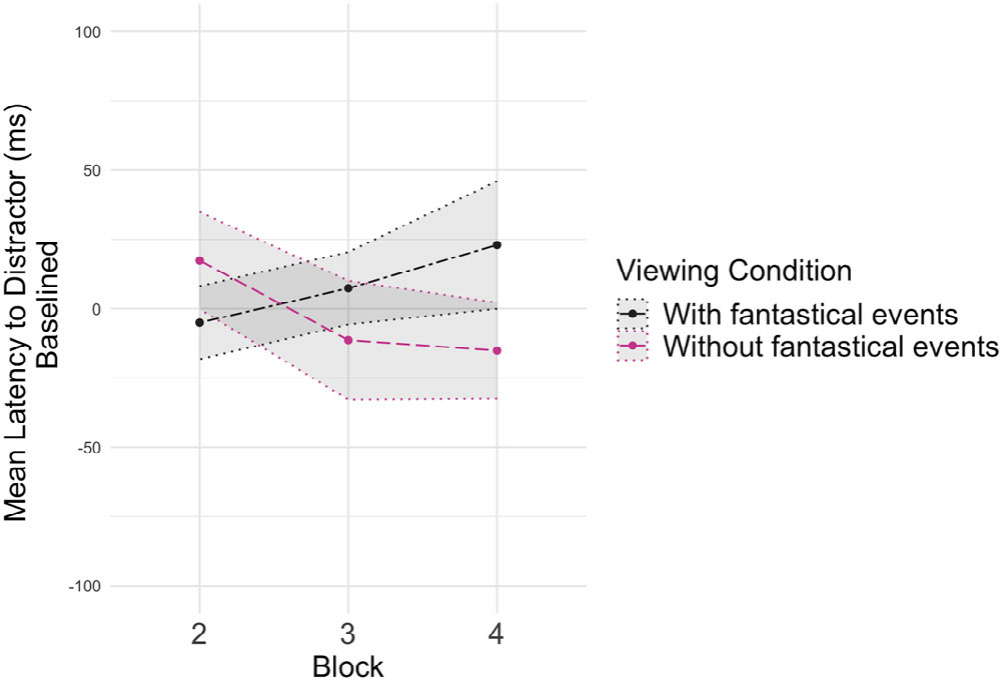
Mean latency to look to the distractor (baselined) in milliseconds for each viewing condition as a function of block. Shaded areas represent the standard error of the mean.

To check whether the lack of a block effect on corrective saccades in the **with fantastical events** condition could be explained by quicker responding to the distractor, we performed an exploratory analysis of the latency to look to the distractor (Figure 5). An initial GEE model with *viewing condition* entered as the predictor of latency to look to the distractor at baseline showed children were quicker to saccade to the distractor before viewing the **with fantastical events** cartoons (mean: 421.57 ms, SD: 61.94) than before the **without fantastical events** cartoons (mean: 456.23 ms, SD: 76.58), see Table 4. These baseline differences can affect behaviour even if the same child is involved in the two conditions. Therefore, baselining before stimulation is a standard approach with time‐series data (eye‐tracking, EEG, etc.). For the baselined latency to look to the distractor, a GEE model with *viewing condition* and *block* entered as predictors of the outcome variable (see Table 2) showed no main effect of viewing condition (*p* = 0.20) or block (*p* = 0.17). The viewing condition*block interaction was also non‐significant (*p* = 0.11) (Figure 5).

**TABLE 4:** Latencies to look to the distractor (before baselining) as mean and standard deviation in each condition by block.

		With fantastical events mean (SD)	Without fantastical events mean (SD)
Saccadic reaction time to the distractor	Block 1 (baseline)	421.57 ms (61.94)	456.23 ms (76.58)
	Block 2	419.16 ms (66.40)	473.05 ms (104.61)
	Block 3	429.95 ms (79.68)	437.67 ms (118.14)
	Block 4	438.85 ms (111.58)	436.58 ms (75.97)

### 3.2 FDs During Cartoon Viewing Are Not Affected by the Presence of Fantastic Events

**FIGURE 6:**
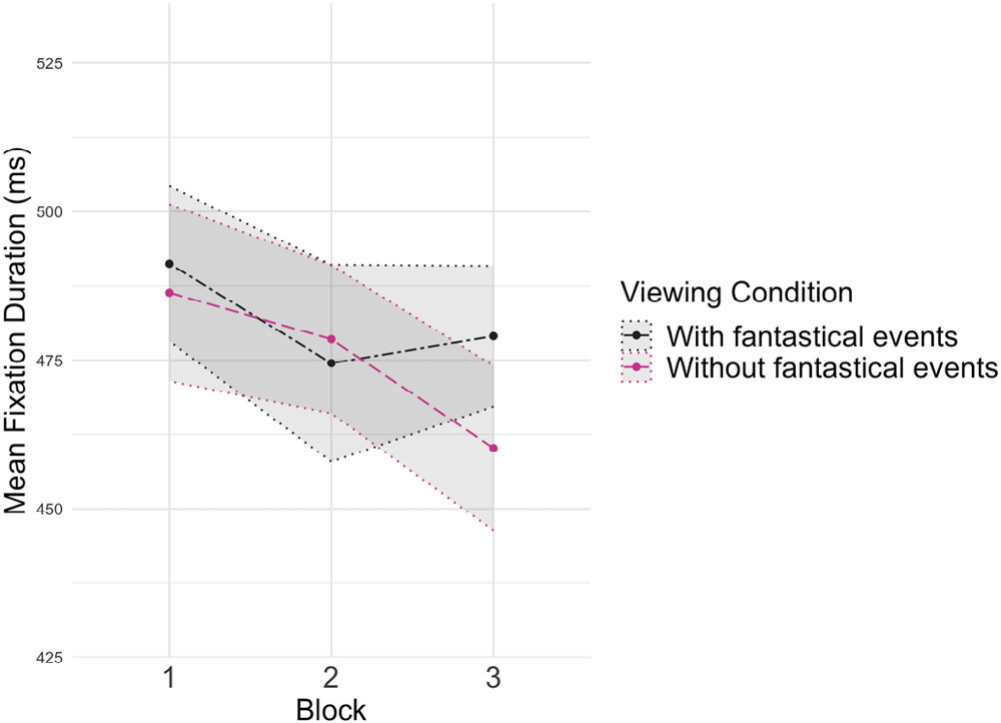
Mean fixation duration in milliseconds for each viewing condition across the three presentations. Shaded areas represent the standard error of the mean.

We next sought to test for exogenous attention differences whilst children viewed the two sets of cartoons (with/without fantastical events). The results from the fixation data are shown in Figure 6; GEE model results are shown in Table 5. A GEE model with condition and block (1–3) entered as predictors of mean FD (ms) showed no significant condition difference (Wald *x* ^2^ = 0.03 (1), *p* = 0.87). Mean FD did not differ by Block (Wald *x* ^2^ = 4.36 (2), *p* = 0.11), and the condition * block interaction was also non‐significant (Wald *x* ^2^ = 2.34 (2), *p* = 0.31).

**TABLE 5:** Summary of generalised estimating equations model effects with viewing condition and block entered as predictors of mean fixation duration during cartoon viewing.

Variables	Wald *x* ^2^ (df), *p* value
Main model with viewing condition and block entered as predictor of mean fixation duration during cartoon viewing	
***Mean fixation duration***	
Viewing condition	0.026 (1), *p* = 0.87
Block	4.361 (2), *p* = 0.11
***Missing (as proportion of all samples)***	***18.43 (1), p < 0.001*** ^a^
Viewing condition * block	2.338 (2), *p* = 0.31

*Note*: Covariates are indicated with italicised text.

^a^ The average change in the outcome variable is associated with a negative change in the co‐variate; for example, longer fixation durations are associated with less missing data.

## 4 Discussion

In this study, we aimed to determine if previously documented EF depletion findings during TV viewing could be attributed solely to the presence of fantastical events in children's TV shows, independent of the typically co‐occurring saliency differences that are associated with exogenous attention. This study also sought to demonstrate EF depletion at a critical age for developing EFs, 18 months of age. First, to measure EF depletion, we examined performance on an anti‐saccade IC task. The reported results suggest that viewing the cartoons **with fantastical events** limited toddlers’ ability to suppress automatic responses to a distractor to make anticipatory looks to a rewarding target. Specifically, toddlers’ ability to make anticipatory looks towards the target location only increased when they viewed the cartoons **without fantastical events**. In comparison, when the same children viewed the cartoons **with fantastical events,** their anticipatory looks did not significantly change over the trials. As a result, they made fewer anti‐saccades. This finding replicates existing EF depletion evidence and, for the first time, shows that fantastical events have a short‐term impact on IC in toddlers. Importantly, by carefully matching the visual saliency of the two sets of clips, we confirm that this short‐term impact can be attributed to the fantastical events, not saliency differences in the stimuli.

Next, we assessed FDs whilst children viewed the two sets of cartoons to understand if there was an exogenous component to this EF depletion effect. Based on existing evidence, we predicted that if the surprising nature of fantastical events increases exogenous attention, this would manifest as shorter FDs when children viewed the cartoons **with fantastical events**. Contrary to previous evidence (Li et al. 2020), we found no condition differences, suggesting that visual saliency differences between the unmatched clips may have partly driven previous effects. However, it is also possible that there was not sufficient sensitivity in FDs as a measure of exogenous attention during viewing. More sophisticated analysis of gaze behaviour (gaze clustering, scan path analysis, or Monte Carlo permutation analysis; Haensel et al. 2020; Mital et al. 2011; Smith and Mital 2013) may be better placed to detect differences in exogenous attention during free viewing. However, these are not commonplace for developmental studies due to the inherent noisiness of toddler gaze data (Wass et al. 2013).

The lack of condition differences during viewing also suggests that the short‐term impact on IC may not have had an exogenous component in the present study. A follow‐up analysis of corrective behaviours on the IC task further supported this. Results from the saccade behaviours indicated that toddlers were less able to reduce their corrective looks (e.g., automatic looks to the distractor followed by a fast anticipatory look to the target) when viewing cartoons **with fantastical events**. In a previous study using the same task (Portugal, Bedford, Cheung, Mason et al. 2021), corrective behaviour was supported by faster orienting. As such, we examined latencies to the distractor to understand if an increase in the speed of orienting, which would indicate increased exogenous attention, could explain their corrective behaviour. However, we failed to detect an increase in their speed of responding to the distractor when viewing the cartoon **with fantastical events**. The non‐significant trend in this viewing condition was in the opposite direction, with a continued lengthening of the latency to the distractor across all blocks. Taken together, when controlling for saliency differences, these results appear to indicate an impact on endogenous control when viewing cartoons **with fantastical events** rather than a bias towards exogenously driven attention.

Fantastical events are cognitively demanding. The violations of expectation that they present likely require a reassessment of knowledge and attempts to resolve the conflict. There may be attention capture towards uninformative areas of the scene, and children may linger on the event if no explanation for the violation is apparent (Perez and Feigenson 2022; Stahl and Feigenson 2015). This may disrupt the semantic thread of the content, further taxing children's cognitive resources. Evidence from adults suggests that sustained mental effort such as this can reduce available cognitive resources and impact behaviours which draw on endogenous control (Borragan et al. 2016; Lorist 2008). The results in the present study appear to align with this evidence. Saccadic control on our IC task can be thought of as a push‐pull between an automatic timer wanting to produce regular short fixations keeping the eyes moving (less cognitively taxing) versus direct control (more cognitively taxing) which seeks to prolong or cancel saccades to modulate processing at fixation and voluntarily direct saccades to task‐relevant locations (Engbert et al. 2005; Findlay and Walker 1999; Nuthmann et al. 2010). Further, these saccadic models (Engbert et al. 2005; Nuthmann et al. 2010) assume that direct control is probabilistic. Saccade programs are cancelled or delayed (extending FDs) by sampling from a probability distribution. The closer to execution the saccade program is, the less likely a cancellation or extension is going to be successful. Thus, to be successful in the task, children need to have sufficient resources to suppress the pull of the gaze to the distractor triggered by the automatic timer. This appears to have been the case on our IC task after children viewed the cartoons **without fantastical events** but not after cartoons **with fantastical events**.

We believe this to be evidence of cognitive fatigue, which impacted endogenous control. However, we note that the apparent absence of differences in FDs during viewing or on latencies to the distractor immediately after viewing cannot entirely discount the possibility that exogenous attention control may have impacted performance. This is because, with these tasks, it is not possible to fully disentangle the endogenous from the exogenous processes. Free‐viewing gaze during both types of clips may have been under the same amount of exogenous control (as is seen in adult gaze clustering irrespective of video viewing tasks; Hutson et al. 2017; Loschky et al. 2015), and the infant adaptation of the anti‐saccade task cannot separate a bias towards the salient distractor from an inability to learn the rewarding nature of the anti‐saccade. We also acknowledge that given the modifications to the anti‐saccade task (e.g., using a motivating reward to encourage anti‐saccade behaviour), caution should be taken in drawing parallels between anti‐saccade behaviours as measured on our task and those measured in older children and adults. A further issue for the modified anti‐saccade task is that it is unclear to what extent habituation to the repeated distractor presentations may alter behaviours. However, prior evidence suggests that a decline in pro‐saccade behaviours with concomitant increases in anti‐saccade behaviours is unlikely to result from habituation (Johnson 1995). In the first study to use a modified anti‐saccade task to assess automatic saccades in infancy, Johnson (1995) found that when the distractor cue was not predictive of the target location, there was no reduction in pro‐saccades. This suggests that the distractor's predictive nature drives reductions in pro‐saccades rather than habituation to the distractor. It will be necessary for future studies to use tasks that allow these processes to be fully disentangled and to chart the potential impact of cognitive fatigue on endogenous control over a longer time course. We also note that the role of attention in EFs continues to be widely debated, with some suggesting that the constructs of WM, IC and attention control likely overlap during early development (e.g., Colombo and Cheatham 2006; Diamond 2002; Ruff and Rothbart 1996). Relatedly, by only employing one measure of IC, we cannot determine whether the impact would generalise to other measures of IC or other core components of EFs, as shown in the prior literature. Further work is necessary to establish whether the present findings can be expanded to encompass broader measures of EFs with differing attentional demands.

An interesting question that arises from these findings is whether there is a point at which children can potentially overcome the challenge of processing these unexpected events through increasing familiarity. We did not find evidence of this with the three repetitions of the cartoons, but this may have been insufficient to elicit familiarity with the cartoons. It seems reasonable to assume that the ability to incorporate novel fantastical events within existing knowledge structures will also vary with age; older children may resolve the conflict induced by fantasy quicker than younger children, for example. Children's cartoons often use repetition to aid comprehension; understanding how familiarity can help or hinder cognitive processes whilst children engage with challenging content would be highly informative for those seeking to provide children with high‐quality interactions with screen media (e.g., content creators and broadcasters). It is also important to note that whilst the cognitive challenge of processing these events appears to be detrimental in the immediate short‐term, there could be a point at which the mental effort exerted to resolve conflicts in the narrative yields a benefit. For example, a study by Kostyrka‐Allchorne et al. (2019) found that response inhibition improved when viewing unrealistic content (e.g., a video of a narrator reading a fantastical story) and protected children from the negative impact of pacing. As such, research should continue to seek to identify any potential benefits as well as negative consequences of engaging with different types of content.

With the present study, we have replicated prior EF depletion findings in toddlers and suggest that the findings may indicate a depletion of resources needed for endogenous control. However, a limitation of this work is that the mechanisms by which EF may be depleted remain unknown. A growing body of evidence from adults suggests metabolic activity in the brain varies with degrees of cognitive effort (e.g., Bruckmaier et al. 2020; Wiehler et al. 2022). With advances in neuroimaging methodology, it will be possible to begin investigating these potential mechanisms in young children. For example, functional and broadband near‐infrared spectroscopy (fNIRS/bNIRS) offer non‐invasive methods for charting functional activation and cerebral metabolic changes during cognitive tasks in developmental populations (Pinti et al. 2020; Siddiqui et al. 2022) and could prove extremely useful for identifying the potential mechanisms which underly the short‐term impact of TV viewing.

It is also important to note that short‐term adverse effects of TV viewing may be dose‐dependent. In the present study, we removed saliency differences between our chosen content, but it is likely that in the media children are exposed to these features co‐occur (e.g., pacing, image complexity, situational properties of time and space; Essex et al. 2022) which may result in additive effects. A further limitation is that our chosen clips contained very little dialogue, which likely lowered the processing demands of both sets of cartoons. It is important to acknowledge that isolating individual properties in this way will not be sufficient to comprehensively understand the potential additive effects in real‐world viewing. As such, it will be crucial for future experimental studies to integrate quantitative tracking of impactful properties across wider selections of TV content. This will be a challenging endeavour as it will require adaptation of current methodologies for analysing screen content (e.g., computational analysis of scene properties with human coding of higher‐level semantic analysis), which can be applied to developmental research.

Finally, the present findings highlight the need to consider how concepts which challenge existing knowledge structures are presented in video content. This may be most pertinent in the context of rapidly changing habits for accessing and engaging with video content (e.g., multi‐platform on‐demand viewing), particularly for young children. Video content generated for online media platforms (YouTube, TikTok, etc.) may be less likely to tailor their content to suit the developmental skills of young viewers and, as such, may amplify any adverse effects (Smith et al. 2023). It is currently unknown whether individual differences in existing media habits may interact with the effects of fantastical content. Our within‐subjects design controlled for such differences, but future studies should directly examine these individual differences. It will be necessary for future studies to investigate the broad range of content young children engage with to build a nuanced view of the impactful properties and to identify content where they may be more prevalent.

To conclude, with the present study, we have shown a detrimental impact of viewing fantastical cartoons on toddlers’ IC, replicating prior evidence and extending this to children under two years of age in a within‐subjects design. We found no evidence of an exogenous component to this short‐term effect, suggesting there may have been a cognitive fatigue arising from processing the challenging novel screen events, which limited toddlers’ endogenous control. Strengths of the study include tightly controlled cartoon stimuli and the use of a within‐subjects repeated measures block design. However, we acknowledge that a modest sample size and a single measure of EFs are limitations of the present work. Future work should seek to replicate these findings, confirm these effects transfer to other core EFs, establish how long the EF depletion may persist, and identify the potential mechanisms that underly the possible cognitive fatigue.

## Author Contributions

C.E., T.G. and T.J.S. developed the study concept. All authors contributed to the study design. Testing and data collection were performed by C.E. Data analysis was performed by C.E. under the supervision of T.G., R.B. and T.J.S. C.E. drafted the manuscript and R.B., T.G. and T.J.S. provided critical revisions. All authors approved the final version of the manuscript for submission.

## Ethics Statement

The study was approved by the Birkbeck Psychological Sciences ethics board and conducted according to the British Psychological Society Code of Ethics and Conduct. Parents provided written informed consent for their children.

## Conflicts of Interest

The authors declare no conflicts of interest.

## Supporting information



Supporting Information

## Data Availability

The data that support the findings of this study are available on request from the corresponding author. The data are not publicly available due to privacy or ethical restrictions.
