# Supplementary material for: Toddlers Viewing Fantastical Cartoons: Evidence of an Immediate Reduction in Endogenous Control Without an Increase in Stimulus‐Driven Exogenous Control
Source: Dev Sci. 2025 Mar 16;28(3):e70008. doi: 10.1111/desc.70008 (PMC11911715; doi:10.1111/desc.70008)
Supplement: Supplementary file 1 — Supporting Information [file DESC-28-e70008-s001.docx]

**Toddlers viewing fantastical cartoons: evidence of an immediate reduction in endogenous control without an increase in stimulus-driven exogenous control.**

Authors: Claire Essex*^1^, Professor Rachael Bedford^2^, Professor Teodora Gliga^3^, Professor Tim J. Smith^1&4^

^1^ Centre for Brain and Cognitive Development, Birkbeck, University of London, UK

^2^ Department of Psychology, Queen Mary, University of London, UK.

^3^ University of East Anglia, Norwich, UK

^4^ Creative Computing Institute, University of the Arts London, UK

*Corresponding Author

Full Name: Claire Essex

Institute: Centre for Brain and Cognitive Development, Department Psychological Sciences, Birkbeck, University of London, Malet Street, London, WC1E 7HX, UK

E-mail: c.essex@bbk.ac.uk

Orcid ID: https://orcid.org/0000-0002-7423-0955

***Supplementary Table S1****. Sample demographics. Continuous data is represented as Mean (standard deviation). Categorical data represented as n (%)*

|  | Sample |
| --- | --- |
| **N** | **36** |
| **Sex** |  |
| **Girls** | 18 (50%) |
| **Boys** | 18 (50%) |
| **Parent/Carer 1 Education** |  |
| **School Leaving** | 0 |
| **College** | 1 (3%) |
| **University** | 15 (42%) |
| **Postgraduate** | 20 (55%) |
| **None/NA** | 0 |
| **Parent/Carer 2 Education** |  |
| **School Leaving** | 1 (3%) |
| **College** | 2 (5%) |
| **University** | 20 (56%) |
| **Postgraduate** | 11 (31%) |
| **None/NA** | 2 (5%) |
| **Index of Multiple Deprivation (IMD) as a proxy for SES** |  |
| **1 (most deprived)** | 0 (0%) |
| **2** | 4 (11%) |
| **3** | 4 (11%) |
| **4** | 7 (19%) |
| **5** | 2 (6%) |
| **6** | 5 (14%) |
| **7** | 3 (8%) |
| **8** | 6 (17%) |
| **9** | 4 (11%) |
| **10 (least deprived)** | 1 (3%) |
| **Age (days)** | 564 (12) |
| **ECBQ** |  |
| **Effortful Control** | 4.23 (.47) |
| **Negative Affect** | 2.93 (.56) |
| **Surgency** | 4.98 (.64) |
| **Media Use in minutes (all media use [video content, music, games, creative apps, internet-based searching] on all devices [TV, mobile devices, computers, games consoles])** | 55 (53) |
|  |  |

***Supplementary Table S2****. Task battery for the lab sample.*

|  | **Study sign-up** | **Prior to Lab Visit 1** | **Lab Visit 1** | **Lab Visit 2** |
| --- | --- | --- | --- | --- |
| **Parent-report surveys completed online** |  |  |  |  |
| **Basic demographic variables including parent/carer's education, date of birth, gender and gestation** | **X** |  |  |  |
| **Media Use Questionnaire** | **X** |  |  |  |
| **Media Diary** |  | **X** |  |  |
| **Very-Short ECBQ + Short Form ECBQ (Attention Scales)** |  | **X** |  |  |
| **Ages & Stages (18 months)** |  | **X** |  |  |
| **Eye-Tracking Based Measures** |  |  |  |  |
| **Visual Search (Pre-Measure only)** |  |  | **x** | **x** |
| **Static Scene Viewing (Pre & Post Measures, 2x Blocks)** |  |  | **x** | **x** |
| **Anti-Saccade (Pre & Post Measures, 4x Blocks)** |  |  | **x** | **x** |
| **Cartoon Viewing (2x conditions, 3x Blocks)** |  |  | **x** | **x** |
| **Word Learning Task (Post Measure only, 2x Blocks)** |  |  | **x** | **x** |
| **Lab-based measures** |  |  |  |  |
| **Lab-Tab Block Orientation - 3 Minute independent play task** |  |  | **x** | **x** |

***Supplementary Table S3.*** *Bayesian Tests of saliency differences*

|  | BF_10_ | Wilcoxon Signed-Rank | R^ | Median | 95%CI | |
| --- | --- | --- | --- | --- | --- | --- |
|  |  |  |  |  | Upper | Lower |
| Low-Level Saliency (Flicker) | 0.65 | 23 | 1 | 0.31 | 0.02 | 0.92 |
| Low-Level Saliency (Flicker)  during fantastical events | 1.03 | 25 | 1 | 0.39 | 0.02 | 1.01 |

***Supplementary Table S4.*** *Distribution of valid trials in the Anti-Saccade Task in each block for the* ***with fantastical events*** *viewing condition.*


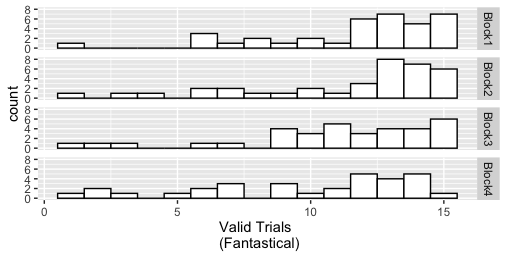


***Supplementary Table S5.*** *Distribution of valid trials in the Anti-Saccade Task in each block for the* ***without fantastical events*** *viewing condition.*

***
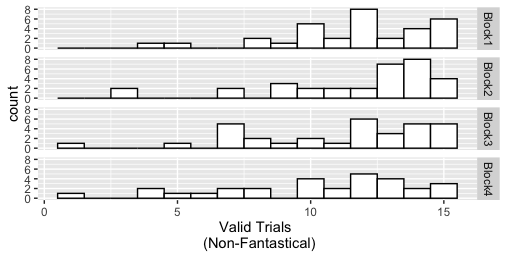
***

***Supplementary Table S6****. Summary of Generalised Estimating Equations model effects at baseline with viewing condition entered as the predictor of saccade behaviour and latency to the distractor.*

| Variables | Wald x^2^ (df), p-value |
| --- | --- |
| Main Model with condition entered as predictor of  saccade behaviour and latency to look to the distractor |  |
| ***Anti-Saccades (as proportion of total valid trials)*** | |
| Viewing condition | .750 (1), p=.39 |
| ***Pro-Saccades (as proportion of total valid trials)*** | |
| Condition | .214 (1), p=.64 |
| ***Corrective Looks (as proportion of total valid trials)*** | |
| Condition | .194 (1), p=.66 |
| ***Latency to look to the Distractor (Saccadic Reaction Time)*** | |
| Condition | 3.63 (1), p=.06 |

***Supplementary Table S7****. Summary table showing the amount of valid data obtained in each block as a function of viewing condition before inclusion criteria was applied (>5 valid trials) and represented as N, Mean number of valid trials (Standard Deviation). The difference between viewing conditions was assessed with a paired samples t-test.*

|  | With fantastical events | Without fantastical events | Paired Sample Comparison |
| --- | --- | --- | --- |
| Block 1 | 35, 12 (3) | 33, 12 (3) | n.s (p=.96) |
| Block 2 | 35, 12 (3) | 33, 12 (3) | n.s (p=.67) |
| Block 3 | 33, 11(4) | 33, 11(4) | n.s (p=.79) |
| Block 4 | 31, 10 (4) | 29, 10 (4) | n.s (p=.30) |

***Supplementary Table S8****. Summary of Generalised Estimating Equations model effects with viewing condition and block (2-4) entered as predictors of number of valid trials. Significant results at p<.05 are shown in bold.*

| Variables | Wald x^2^ (df), p-value |
| --- | --- |
| Main model with viewing condition and block entered as predictor of number of valid trials |  |
| ***Mean number of valid trials*** | |
| Viewing condition | .16 (1), p=.69 |
| **Block** | **7.15 (2), p=.03** |
| Viewing condition * block | .22 (2), p=.90 |

***Supplementary Table S9.*** *Summary of saccade behaviours without baseline correction once inclusion criteria was applied (>5 valid trials). Saccade behaviours are represented as the mean proportion (standard deviation) as a function of saccade type (Anti, Pro, Corrective), block, and viewing condition.*

|  |  | With fantastical events | | |  | Without fantastical events | | |
| --- | --- | --- | --- | --- | --- | --- | --- | --- |
| Block | N | Anti-Saccade | Pro-Saccade | Corrective Look | N | Anti-Saccade | Pro-Saccade | Corrective Look |
| 1 | 34 | 0.31 (.23) | 0.36 (.24) | 0.27 (.19) | 31 | 0.26 (.27) | 0.39 (.27) | 0.29 (.26) |
| 2 | 32 | 0.61 (.27) | 0.18 (.20) | 0.17 (.14) | 31 | 0.58 (.27) | 0.16 (.16) | 0.22 (.22) |
| 3 | 30 | 0.56 (.25) | 0.14 (.16) | 0.15 (.11) | 31 | 0.63 (.27) | 0.11 (.17) | 0.17 (.18) |
| 4 | 26 | 0.61 (.27) | 0.15 (.19) | 0.15 (.19) | 24 | 0.74 (.22) | 0.09 (.14) | 0.13 (.13) |
